# Influenza A Virus NS1 Protein Promotes Efficient Nuclear Export of Unspliced Viral M1 mRNA

**DOI:** 10.1128/JVI.00528-17

**Published:** 2017-07-12

**Authors:** Carina F. Pereira, Eliot K. C. Read, Helen M. Wise, Maria J. Amorim, Paul Digard

**Affiliations:** a Division of Virology, Department of Pathology, University of Cambridge, Tennis Court Road, Cambridge, United Kingdom; b The Roslin Institute, University of Edinburgh, Easter Bush, United Kingdom; c Cell Biology of Viral Infection, Instituto Gulbenkian de Ciência, Oeiras, Portugal; St. Jude Children's Research Hospital

**Keywords:** influenza A virus mRNA export, NXF1, NS1, nuclear import/export

## Abstract

Influenza A virus mRNAs are transcribed by the viral RNA-dependent RNA polymerase in the cell nucleus before being exported to the cytoplasm for translation. Segment 7 produces two major transcripts: an unspliced mRNA that encodes the M1 matrix protein and a spliced transcript that encodes the M2 ion channel. Export of both mRNAs is dependent on the cellular NXF1/TAP pathway, but it is unclear how they are recruited to the export machinery or how the intron-containing but unspliced M1 mRNA bypasses the normal quality-control checkpoints. Using fluorescent *in situ* hybridization to monitor segment 7 mRNA localization, we found that cytoplasmic accumulation of unspliced M1 mRNA was inefficient in the absence of NS1, both in the context of segment 7 RNPs reconstituted by plasmid transfection and in mutant virus-infected cells. This effect was independent of any major effect on steady-state levels of segment 7 mRNA or splicing but corresponded to a ∼5-fold reduction in the accumulation of M1. A similar defect in intronless hemagglutinin (HA) mRNA nuclear export was seen with an NS1 mutant virus. Efficient export of M1 mRNA required both an intact NS1 RNA-binding domain and effector domain. Furthermore, while wild-type NS1 interacted with cellular NXF1 and also increased the interaction of segment 7 mRNA with NXF1, mutant NS1 polypeptides unable to promote mRNA export did neither. Thus, we propose that NS1 facilitates late viral gene expression by acting as an adaptor between viral mRNAs and the cellular nuclear export machinery to promote their nuclear export.

**IMPORTANCE** Influenza A virus is a major pathogen of a wide variety of mammalian and avian species that threatens public health and food security. A fuller understanding of the virus life cycle is important to aid control strategies. The virus has a small genome that encodes relatively few proteins that are often multifunctional. Here, we characterize a new function for the NS1 protein, showing that, as well as previously identified roles in antagonizing the innate immune defenses of the cell and directly upregulating translation of viral mRNAs, it also promotes the nuclear export of the viral late gene mRNAs by acting as an adaptor between the viral mRNAs and the cellular mRNA nuclear export machinery.

## INTRODUCTION

Influenza A virus (IAV) has a genome constituted of eight single-stranded, negative-sense RNA molecules, each separately encapsidated into viral ribonucleoprotein (vRNP) particles with one copy of the viral PB1-PB2-PA (3P) heterotrimeric RNA polymerase and multiple copies of a nucleoprotein (NP) (1). These RNPs are the templates for transcription and replication of the genome (the latter by a cRNA replicative intermediate), which occurs in the host cell nucleus. Use of this cellular compartment provides access to the mRNA splicing machinery and potentially avoids cytoplasmic viral RNA sensors such as RIG-I but introduces the need for nuclear export of both replicated genomic vRNA (as vRNPs) and viral mRNA. Nuclear export of IAV vRNPs is well characterized; the CRM1 cellular pathway is accessed by the participation of two further viral proteins, M1 and NS2/NEP, with NS2 acting as an adaptor protein between vRNP-bound M1 and CRM1 itself (2, 3). The mechanism underlying IAV mRNA export, however, remains less well understood, which is further complicated by the fact that there are different types of transcripts to consider: intronless (from segments 1 to 6), intron-containing but unspliced (segments 7 and 8; mRNAs encoding M1 and NS1, respectively), and their fully spliced counterparts (for segment 7; either mRNA2 encoding M2 or a so far hypothetical 9-mer peptide encoded by mRNA3 and NS2 from spliced segment 8 mRNA [4]). These mRNAs can additionally be divided into early (segments 1 to 3, 5, and unspliced 8) and late (segments 4, 6, unspliced 7, and the two spliced transcripts) classes, according to the expression kinetics of the proteins they encode (4, 5), raising the possibility that different strategies might be used for specific mRNAs. All mRNA transcripts nevertheless start with a host-derived ^7m^GpppG_m_ cap structure derived from cellular pre-mRNAs by the process of “cap-snatching” (1), which provides structural identity between the 5′ ends of viral and cellular mRNAs.

Several of the viral late gene mRNAs have been shown to be retained in the nucleus in the presence of inhibitors of RNA polymerase II (Pol II) (6–8), suggesting that they normally use a cellular pathway for nuclear export. Subsequent work has identified this as the cellular NXF1 (TAP)-dependent pathway, the route by which the majority of cellular mRNAs reach the cytoplasm (9, 10). RNA interference (RNAi) silencing of NXF1 in human embryonic kidney 293 cells inhibited viral gene expression and overall replication (11) and was subsequently shown to reduce nuclear export of all influenza virus mRNAs tested, albeit with a gradient of sensitivity in which the M1 mRNA was the most sensitive and NP mRNA was the least sensitive (12, 13). Furthermore, IAV mRNAs from segments 2, 4, 5, 6, and 7 have been shown to coprecipitate with the cellular nuclear cap-binding protein and/or NXF1 (13–15), further confirming the use of the cellular mRNA export pathway.

Cellular mRNAs are recruited to the NXF1 pathway cotranscriptionally by the deposition of various cellular factors (the transcription-export or TREX complex) onto the nascent mRNA after addition of the 5′ cap structure as well as during the removal of introns (16–19). The Aly component of TREX then hands over the mRNA to NXF1 and its cofactor p15, which then interact with the nuclear pore to direct export of the transcript (20). However, since all influenza virus mRNAs are synthesized by the viral polymerase rather than by RNA Pol II and since most either do not contain introns or are exported with unprocessed introns, it is not clear how they are recruited to the NXF1 pathway. Herpesviruses have solved the problem of exporting intronless viral mRNAs by providing a viral adaptor protein to interact with Aly and thus recruit the TREX complex components needed for export (21). The IAV NS1 protein has been hypothesized to perform an analogous function, based on its known interactions with various cellular mRNA processing and export factors as well as with viral mRNAs (22). Consistent with this, a recent study defined a role for NS1 in recruiting segment 7 mRNA to nuclear speckles for splicing and also for subsequent nuclear export (23). To further define the export pathways used by IAV mRNAs, we examined the minimum requirements for nuclear export of viral mRNAs transcribed by reconstituted RNPs, focusing on segment 7 mRNAs. We found that they are largely retained in the nucleus in the absence of the viral NS1 protein and that while wild-type (WT) NS1 bound both viral mRNA and NXF1 and promoted the interaction of segment 7 mRNA with NXF1, mutant NS1 proteins that had lost these activities failed to support efficient export of viral mRNA. Furthermore, hemagglutinin (HA) mRNA was inefficiently exported in the absence of a fully functional NS1 protein. Thus, we conclude that the NS1 protein acts as an adapter molecule to direct viral late gene mRNAs to the cellular nuclear export machinery.

## RESULTS

### Synthetic viral late gene mRNAs are inefficiently exported in the absence of NS1.

Influenza A virus transcription occurs in the nucleus of infected cells, which necessitates that viral mRNAs be exported to the cytoplasm. This is achieved at least in part by use of the main cellular mRNA NXF1/TAP-dependent export pathway (8, 11–13, 15, 23). However, transcripts from individual segments show differential requirements for the cellular export machinery (8, 12, 13), and it is not clear how the viral mRNAs are fed into the cellular export pathway. To pursue the hypothesis that a viral polypeptide acts as an adapter between the viral transcription machinery and the cellular export pathway, we compared the localization of individual viral mRNAs in the context of virus infection and in the context of an RNP reconstitution system, reasoning that the latter approach might reveal a role for a viral component other than the minimal requirements of the three polymerase subunits and NP (3PNP) needed for transcription in a minireplicon assay. We focused on segment 7 mRNAs because prior work had indicated a strong dependency on the cellular NXF1 pathway for their nuclear export (8, 12, 15).

**FIG 1:**
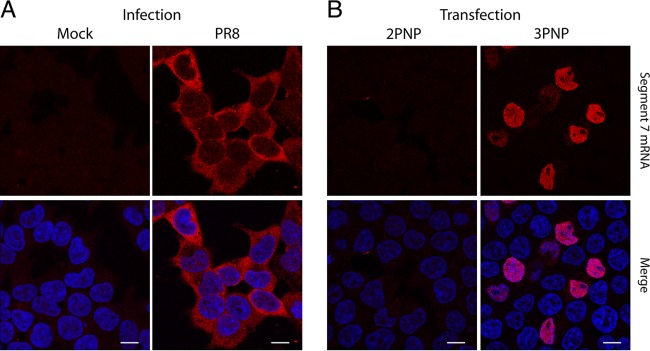
Localization of segment 7 mRNA in infected and transfected cells. 293T cells were infected or mock infected with Cambridge PR8 at an MOI of 5 and fixed at 6 h p.i. (A) or transfected with plasmids to reconstitute RNPs (3PNP) containing segment 7 vRNA or with a negative-control set lacking PB2 (2PNP) and fixed 24 h later before. Cells were then stained for positive-sense segment 7 RNA by FISH (red) or for DNA (4′,6′-diamidino-2-phenylindole; blue) and imaged by confocal microscopy (B). Single optical slices are shown. Scale bar, 10 μm.

When segment 7 mRNA localization was observed by fluorescence *in situ* hybridization (FISH) of virus-infected 293T cells at 6 h postinfection (p.i.) (using a probe complementary to both unspliced M1 and spliced M2 mRNAs), the majority of the transcripts were cytoplasmic (Fig. 1A), as expected (8, 12, 23). Time course experiments showed substantial cytoplasmic accumulation of segment 7 mRNA from as early as 4.5 h p.i. (data not shown). However, when cells were transfected with 3P and NP expression plasmids and a plasmid encoding segment 7 vRNA under an RNA polymerase I promoter (Pol I) to reconstitute segment 7 RNPs, the transcripts showed marked (although not total) nuclear retention at 24 h posttransfection (Fig. 1B). The negative controls for both infection (mock-infected cells) and transfection (lacking the PB2 subunit of the polymerase [2PNP]) gave no significant signal, showing the specificity of the probe used. Thus, segment 7 mRNAs were not exported efficiently in the RNP reconstitution system, suggesting the normal involvement of a viral factor coming from a gene not included in the minimal set needed to recreate an RNP.

**FIG 2:**
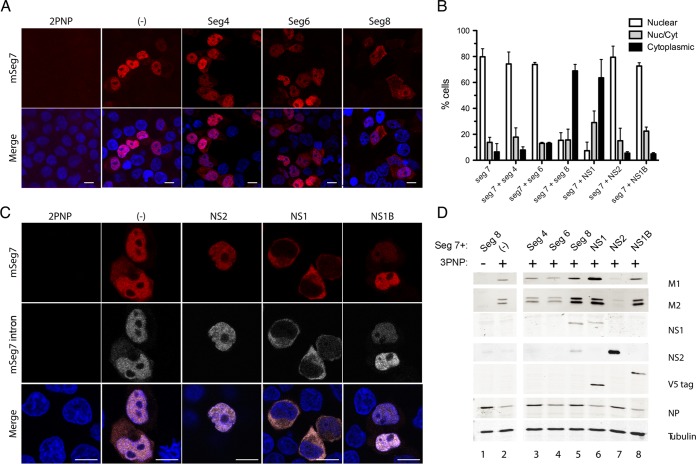
NS1 promotes cytoplasmic accumulation of M1 mRNA. 293T cells were transfected with plasmids to reconstitute RNPs (3PNP) containing segment 7 vRNA or with a negative-control set lacking PB2 (2PNP or −) as well as with other segments or plasmids expressing NS1 or NS2 only or NS1 from influenza B virus (NS1B) and fixed 24 h later. Cells were then stained for positive-sense segment 7 RNA (red) (A and C) or the intronic sequence of M1 (gray) by FISH or for DNA (4′,6′-diamidino-2-phenylindole; blue) and imaged by confocal microscopy (C). Single optical slices are shown. Scale bar, 10 μm. (B) Individual cells were scored as to whether segment 7 mRNA staining was predominantly nuclear, cytoplasmic, or mixed. The means ± standard errors of the means from two to five independent experiments are plotted. (D) Cell lysates were analyzed by Western blotting for the indicated antigens. mSeg7, segment 7 mRNA.

Next, the transfected minimal segment 7 transcriptional unit was supplemented with additional Pol I plasmids that expressed each of the missing vRNAs (segments 4, 6, and 8), and segment 7 mRNA localization was observed as before by FISH. Again, positive-sense transcripts from reconstituted segment 7 RNPs alone were largely nuclear (Fig. 2A). The addition of either segment 4 or segment 6 (and thus the expected expression of HA or neuraminidase [NA], respectively) did not alter segment 7 mRNA localization. Addition of segment 8 did substantially alter the staining pattern, however, with many more cells showing markedly greater amounts of cytoplasmic staining. When replicate experiments were scored for the number of cells showing predominantly nuclear, predominantly cytoplasmic, or a mixed pattern of segment 7 mRNA localization, the addition of segment 8, but not segment 4, caused a clear shift toward cytoplasmic localization (Fig. 2B), indicating that a segment 8 gene product promotes segment 7 mRNA export.

Segment 8 of A/PR/8/34 (PR8) encodes two identified proteins: NS1, produced from the unspliced mRNA transcript, and NS2/NEP, from a spliced mRNA (24, 25). To distinguish between the effects of NS1 and NS2, plasmids expressing either influenza A virus NS1 or influenza A virus NS2 proteins were transfected together with segment 7 and 3PNP. As a further control, we also tested NS1 from influenza B virus (NS1B). In addition, because segment 7 produces spliced and unspliced mRNAs (26, 27), the cells were hybridized with an intron-specific probe specific for M1 mRNA as well as with the pan-segment 7 probe. Both probes indicated a predominantly nuclear localization for all detectable positive-sense segment 7 RNA species when segment 7 RNPs were reconstituted alone (Fig. 2C). NS2 protein alone was not capable of rescuing segment 7 mRNA export as the mRNA was visibly still retained in the nuclei of most cells (Fig. 2B and C). Similarly, the addition of NS1B did not result in segment 7 mRNA export. In contrast, addition of NS1 from IAV had a clear effect on mRNA localization as the majority of cells now displayed mostly cytoplasmic fluorescence with both segment 7 probes.

Protein expression of the segment 7 and 8 gene products was analyzed by Western blotting, confirming the expression of NS1, NS2, and NS1B in the expected samples (Fig. 2D, lanes 5 to 8; note the presence of V5 epitope tags on NS1 and NS1B proteins). NP levels were comparable between samples, suggesting similar transfection levels, while an examination of tubulin levels confirmed equal gel loading. M1 and M2 proteins were not detected in the negative control where segment 7 transcription was blocked by the omission of PB2 (Fig. 2D, lane 1). Otherwise, M1 and M2 proteins (the latter running as a doublet, possibly because of posttranslational modification [28]) were detected under all conditions where a segment 7 RNP was reconstituted (lanes 2 to 8). Notably, however, the expression levels for M1 and M2 were higher when either segment 8 or NS1 was added (lanes 5 and 6). This increase in M1 and M2 accumulation in the presence of NS1 is consistent with the more efficient release of segment 7 mRNA to the cytoplasm seen by FISH, as well as with the ability of NS1 to increase translation of viral mRNAs.

**FIG 3:**
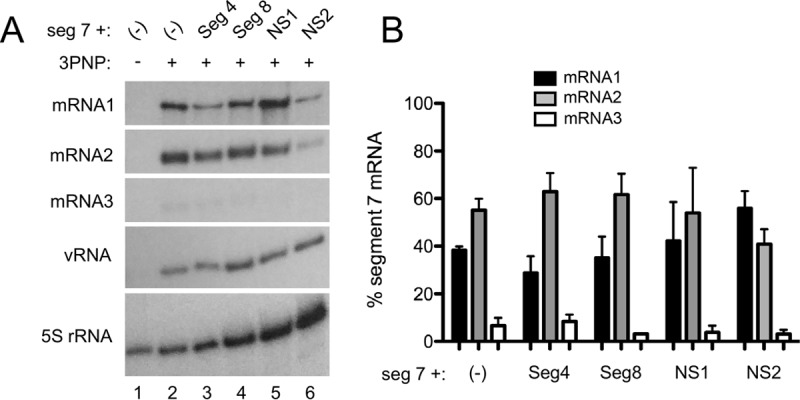
Accumulation of segment 7 mRNA species in RNP reconstitution assays. (A) 293T cells were transfected with plasmids to recreate segment 7 RNPs or, as a negative control, with a 2PNP combination lacking PB2 either alone (−) or along with other viral components as labeled. Twenty-four hours later, total cellular RNA was extracted and analyzed by radioactive reverse transcriptase primer extension followed by urea-PAGE and autoradiography with primers specific for segment 7 mRNAs or cellular 5S rRNA. (B) Replicate experiments were quantified by densitometry, and the percentage of total segment 7 mRNA for each of the three species was determined. Data are the means ± standard deviations (*n* = 3).

In some (but not all) studies, NS1 expression has been found to affect the extent of segment 7 splicing (23, 29–31). It was therefore conceivable that, if the M1 and M2 mRNAs have intrinsic differences in transport efficiency, NS1 could indirectly promote segment 7 mRNA export by changing the balance between spliced and unspliced products. To test this hypothesis, segment 7 mRNA splicing was analyzed using radiolabeled-primer reverse transcription. In our A/PR/8/34 (PR8)-based system, M1 and M2 mRNAs accumulated to approximately equal amounts in the absence of NS1, while mRNA3 formed a minority species (Fig. 3A). The levels of genomic vRNA were also reasonably consistent. However, expression of NS1 had variable and generally modest effects on the overall levels of segment 7 splicing, with mRNA2 remaining slightly more abundant, on average, than mRNA1 (Fig. 3B). The most consistent effect, of a slight suppression of splicing, was in fact seen with NS2, which did not promote cytoplasmic accumulation of the transcripts. In all cases, NS1 (or NS2) expression changed the relative abundance of the individual segment 7 mRNA species by less than 2-fold. Overall, therefore, this indicated that the PR8 NS1 protein promotes efficient nuclear export of segment 7 mRNAs without major effects on their differential splicing.

**FIG 4:**
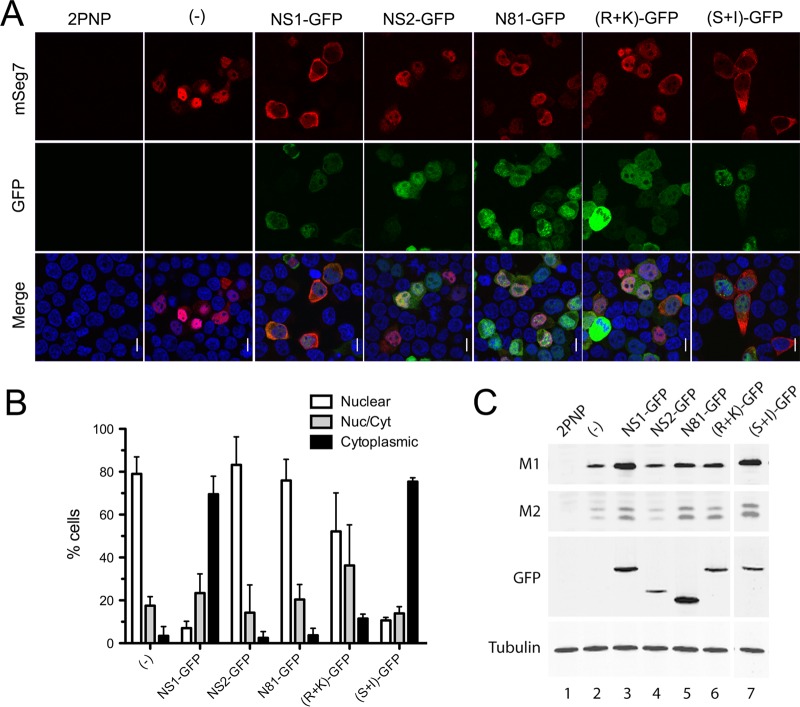
Ability of GFP-tagged NS polypeptides to support segment 7 mRNA export. (A) 293T cells were transfected with plasmids to reconstitute RNPs containing segment 7 vRNA or with a negative-control set lacking PB2 (2PNP) as well as with plasmids expressing the indicated GFP-tagged proteins and fixed 24 h later before being stained for GFP (green), positive-sense segment 7 RNA (red, and DNA (4′,6′-diamidino-2-phenylindole; blue) and imaged by confocal microscopy. Single optical slices are shown. Scale bar, 10 μm. (B) Individual cells were scored as to whether segment 7 mRNA staining was predominantly nuclear, cytoplasmic, or mixed. Values are the means ± standard deviations from two to five independent experiments. (C) Parallel samples were processed by Western blotting for the indicated polypeptides.

NS1 protein has two functional domains, an effector domain and an RNA-binding domain, each associated with several functions (32). To assess whether the ability of NS1 to promote segment 7 mRNA export was intrinsic to one of the domains, NS1 mutants were generated fused with a green fluorescent protein (GFP) tag. As before, each of the NS1-GFP mutants was added separately to the segment 7 RNP reconstitution assay, and segment 7 mRNA localization was analyzed by FISH 24 h later. As expected, segment 7 mRNA could not be detected in the 2PNP, the negative control, and when segment 7 mRNA was transcribed in the absence of any additional non-RNP influenza A proteins, it was found in the nucleus, as well as when it was made in the presence of NS2-GFP (Fig. 4A and B). In contrast, when NS1-GFP was also transfected, the mRNA was efficiently exported to the cytoplasm, indicating that the addition of a GFP tag did not block the export-promoting activity of NS1. An NS1-GFP mutant, in which cleavage/polyadenylation specificity factor 30 (CPSF)-inhibitory activity had been restored by appropriate mutation of effector domain residues S103 and I106 [(S+I)-GFP] (33, 34), also promoted the efficient export of segment 7 mRNA. However, when a mutant NS1 consisting of only the RNA-binding domain (N81-GFP; where a stop codon was inserted at codon 82) was cotransfected, the mRNA was found largely retained in the nucleus. Similarly, an RNA-binding domain mutant (R+K)-GFP with charge-to-alanine mutations in residues R38 and K41 was not capable of releasing segment 7 mRNA from the nucleus of the majority of transfected cells. Thus, both functional domains of NS1 protein are required to promote export of segment 7 mRNA. Expression of each GFP construct was confirmed by confocal microscopy (Fig. 4A) as well as by Western blot analyses (Fig. 4C), with the latter approach also showing increased expression of M1 and M2 polypeptides in the presence of the fully export-competent NS1 polypeptides and an intermediate phenotype from the N81 or R+K mutant.

### NS1 is required for efficient M1 mRNA export in infected cells.

**FIG 5:**
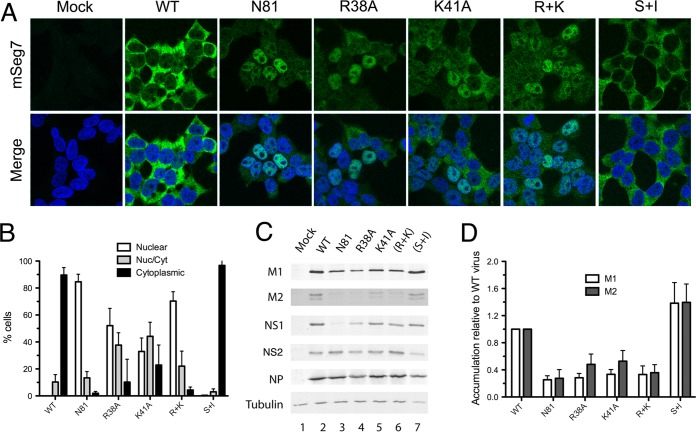
Effect of NS1 mutations on segment 7 mRNA localization in infected cells. (A) 293T cells were infected with the indicated viruses at an MOI of 5, and at 6 h p.i. they were stained for segment 7 mRNA by FISH (green) and for DNA (4′,6′-diamidino-2-phenylindole; blue) before confocal imaging. Single optical slices are shown. Scale bar, 10 μm. (B) Individual cells were scored as to whether segment 7 mRNA staining was predominantly nuclear, cytoplasmic, or mixed. Values are the means ± standard errors of the means from three to six independent experiments. (C) Cell lysates were analyzed by Western blotting for the indicated antigens. (D) M1 and M2 accumulation from replicate experiments was quantified and expressed as a ratio relative to NP expression. Values plotted are normalized to the ratio seen with WT virus and are the means ± standard errors of the means of three independent experiments.

To test whether NS1 was required for efficient segment 7 mRNA export in the context of a viral infection, NS1 mutant viruses were generated by reverse genetics, with none of the mutations affecting the NS2 gene. Further single-residue RNA-binding domain mutants (NS1-R38A and NS1-K41A) were generated in addition to the double mutant NS1-(R+K). Cells were infected with WT PR8 or an NS1 mutant virus, and segment 7 mRNA localization was observed by FISH at 6 h p.i. Segment 7 mRNA was not detected in mock-infected cells and was found in the cytoplasm in WT-infected cells, as observed previously (Fig. 5A). A similar outcome was obtained with the CPSF-binding site mutant virus NS1-(S+I). However, in agreement with the findings from RNP reconstitution assays, neither the R+K RNA-binding domain mutant nor the N81 effector domain mutant supported normal segment 7 mRNA export. When replicate experiments were imaged and scored for the proportion of cells showing nuclear retention of segment 7 mRNA, around 70 to 80% of cells infected with the R+K or N81 mutant showed this pattern, in contrast to the overwhelmingly cytoplasmic phenotype of WT- or S+I mutant-infected cells (Fig. 5B). Analysis of single-residue RNA-binding mutants showed an intermediate effect, with some cells supporting apparently normal mRNA export and others showing nuclear retention (Fig. 5A and B). Western blot analysis of infected cell lysates confirmed expression of all NS1 polypeptides with the exception of the truncated protein produced by the NS1-N81 mutant, which could not be detected by the effector domain-specific antiserum used here (Fig. 5C and data not shown) (the trace amount of apparently full-length NS1 detectable in lane 3 likely reflects low levels of reversion in the virus stock). NP and NS2 expression levels were consistent between all the viruses, but the accumulation of segment 7-derived polypeptides showed a clear correlation with the localization of the mRNA, with efficient nuclear export leading to higher levels of M1 and M2 synthesis (Fig. 5C). Quantification of M1 and M2 accumulation from replicate experiments showed that poor nuclear export of segment 7 mRNA led to around a 3- to 5-fold reduction in the quantity of M1 relative to that of NP and 2- to 3-fold reductions in the amount of M2 (Fig. 5D). Thus, in infection as well as in transfection, a functional NS1 protein is required to promote efficient nuclear export and expression of segment 7 mRNA.

**FIG 6:**
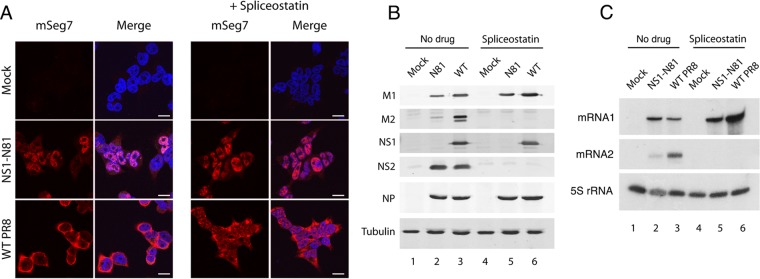
Effect of spliceostatin A on segment 7 mRNA localization. (A) 293T cells were infected with reverse genetics PR8 virus at an MOI of 5 and at 6 h p.i. treated or mock treated with 100 μg/ml spliceostatin A. At 6 h p.i. samples were fixed and stained by FISH for segment 7 mRNA (red) or by 4′,6′-diamidino-2-phenylindole for DNA (blue) and imaged by confocal microscopy. Single optical slices are shown. Scale bar, 10 μm. (B) Cell lysates were examined by Western blotting for the indicated polypeptides. (C) Total cellular RNA was analyzed by radioactive primer extension, urea-PAGE, and autoradiography for the indicated RNA species.

As a further test of the hypothesis that NS1 might promote export of segment 7 mRNA through its effects on the cellular splicing machinery, we asked whether a drug that inhibits splicing affected mRNA export in the presence or absence of a functional NS1 protein. The SF3b inhibitor, spliceostatin A, has been shown to potently inhibit pre-mRNA splicing and consequently suggested to allow the passage of intron-containing transcripts to the cytoplasm (35). Cells were infected with either WT or NS1-N81 virus or mock infected, and duplicate samples were treated with spliceostatin A. At 6 h p.i., segment 7 mRNA cellular localization was determined by FISH. As before, the mRNA was cytoplasmic in WT infection and largely retained in the nucleus after infection by the N81 virus, but neither outcome was changed by the addition of the drug (Fig. 6A). Analysis of viral protein expression by Western blotting confirmed that M1 and NS1 (produced from the unspliced transcripts of segment 7 and 8, respectively) were expressed with or without drug but that spliced products M2 and NS2 were detected only in non-drug-treated infected cells (Fig. 6B, compare lanes 2 and 3 with lanes 5 and 6). Analysis of segment 7 mRNA accumulation by primer extension further confirmed that the drug blocked production of spliced mRNA2 (Fig. 6C). Thus, the mRNA export function of NS1 is independent of mRNA splicing, either as a positive or negative factor.

### NS1 interacts with NXF1 and viral mRNAs to promote their export.

**FIG 7:**
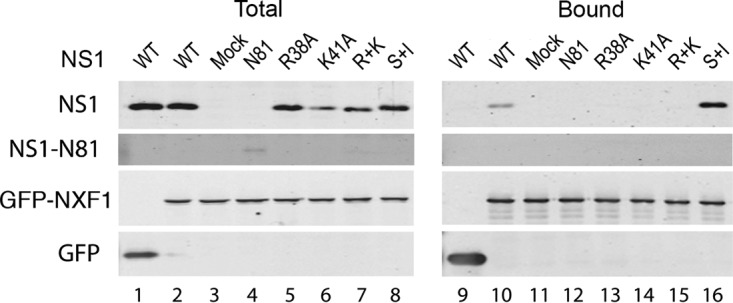
Interaction between NXF1 and NS1. 293T cells were transfected with plasmids encoding GFP or GFP-NXF1 and 48 h later either mock infected or infected with the indicated NS1 mutant viruses. Cells were harvested at 6 h p.i., and cell lysates were examined by Western blotting for the indicated proteins before (Total) or after (Bound) fractionation over GFP-Trap agarose.

We next tested the hypothesis that NS1 protein acted as an adaptor protein to deliver the viral mRNA to the cellular mRNA export machinery. This hypothesis is consistent with the dependence of segment 7 transcripts on the cellular NXF1 pathway for export (12) as well as with interactions between influenza A virus mRNAs and both NS1 and NXF1 and between NS1 and NXF1 themselves (13, 15, 36, 37). First, we examined whether mutant NS1 proteins that failed to promote segment 7 mRNA export bound NXF1. 293T cells were transfected with either GFP or GFP-NXF1 and 48 h later mock infected or infected with the WT or the various NS1 mutant viruses. At 6 h p.i. cells were collected and lysed, and the supernatants were subjected to GFP-Trap pulldown. Western blot analyses of total and bound fractions showed that GFP-NXF1 and GFP were expressed as expected and bound well to the affinity matrix (Fig. 7). NS1 was present in all infected samples, including that with the truncated N81 protein although detection of this last polypeptide required use of an RNA-binding domain-specific antiserum and was inefficient (Fig. 7, lanes 1 and 2 and lanes 4 to 8). Consistent with previous reports (13, 36), WT NS1 coprecipitated with NXF1, as did the NS1 S+I mutant (lanes 10 and 16). However, none of the NS1 RNA-binding domain mutants bound to detectable levels (lanes 13 to 15). Similarly, the effector domain-deleted N81 protein was not apparent in the bound fraction (lane 12), suggesting that (within the limits of detection of the antibody) the truncated protein did not bind NXF1. Thus, there was a good correlation between the ability of NS1 to promote segment 7 mRNA export and its ability to bind NXF1.

**FIG 8:**
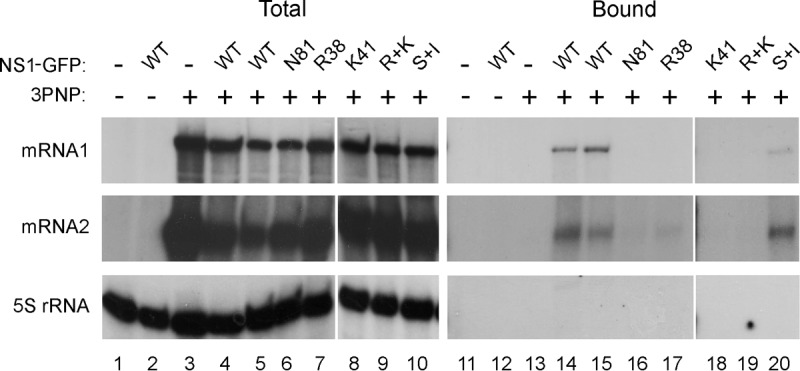
Interaction between segment 7 mRNAs and NS1. 293T cells were transfected with plasmids to recreate segment 7 RNPs (3PNP +) or, as a negative control, with a 2PNP combination (3PNP −) along with the indicated GFP-NS1 polypeptides or with GFP only (NS1-GFP −). Forty-eight hours later, total cellular RNA was extracted and analyzed by radioactive reverse transcriptase primer extension followed by urea-PAGE and autoradiography with primers specific for segment 7 mRNAs or cellular 5S rRNA.

Previous work has shown that NS1 binds segment 7 mRNA *in vitro* and coprecipitates it from infected cells (15, 37, 38). We therefore tested whether the strength of this interaction correlated with the ability of NS1 mutants to promote efficient export of M1 mRNA. First, we used an RNP reconstitution assay to recreate segment 7 RNPs, with or without the addition of WT or mutant GFP-NS1 polypeptides, before fractionating cell lysates over GFP-Trap beads and analyzing the amounts of bound segment 7 mRNA. Examination of aliquots of total cell lysate showed the expected presence of M1 and M2 mRNA species in all samples transfected with all four RNP polypeptides but not in 2P (PB1 and PA) control samples (Fig. 8, lanes 1 to 10). No detectable mRNA coprecipitated with GFP, but easily detectable amounts of both mRNA1 and mRNA2 bound to duplicate samples cotransfected with WT GFP-NS1 or (S+I)-GFP-NS1 (lanes 14, 15, and 20). However, the export-incompetent N81, R38, K41, and R+K mutants all failed to detectably bind M1 mRNA and/or bound greatly reduced amounts of M2 mRNA (lanes 16 to 19).

**FIG 9:**
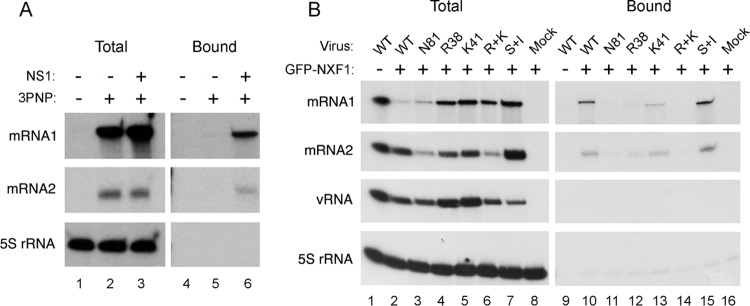
NS1 promotes the interaction of segment 7 mRNA with NXF1. (A) 293T cells were transfected with plasmids to recreate segment 7 RNPs (3PNP +) or, as a negative control, with a 2PNP combination (3PNP −) along with GFP-NXF1 and with or without NS1 as labeled. Forty-eight hours later, total cellular RNA was extracted and analyzed by radioactive reverse transcriptase primer extension followed by urea-PAGE and autoradiography with primers specific for segment 7 mRNAs or cellular 5S rRNA. (B) Cells were transfected with GFP-NXF1 (+) or with GFP alone (−) and 48 h later infected or mock infected with the indicated viruses at an MOI of 10. Total RNA was extracted at 6 h p.i. and analyzed as described for panel A except that a primer specific for segment 7 vRNA was also included.

NXF1 has also been shown to bind M1 mRNA in WT virus-infected cells (15). We therefore used the RNP reconstitution system to ask if (as predicted by the adaptor hypothesis) NS1 facilitated this interaction. 293T cells were transfected with the plasmids needed to recreate segment 7 RNPs (or with a 2P negative control) along with GFP-NXF1 and, additionally, with or without NS1. Forty-eight hours later, cells were lysed, and segment 7 mRNA accumulation was examined by primer extension before or after GFP-Trap affinity purification. Abundant quantities of M1 mRNA and lesser amounts of spliced mRNA2 were present in the total cell lysates from the 3PNP but not the 2P control samples, while examination of cellular 5S rRNA confirmed the extraction of equal cell numbers (Fig. 9A, lanes 1 to 3). No detectable M2 mRNA (or 5S rRNA) and only trace quantities of M1 mRNA copurified with GFP-NXF1 in the absence of NS1 (lane 5). However, both viral mRNAs were readily detectable in samples containing GFP-NXF1 and NS1 (lane 6). Thus, NS1 promotes the stable interaction of NXF1 and segment 7 mRNA.

Next, we correlated the ability of mutant NS1 proteins to promote NXF1-segment 7 mRNA interactions with their mRNA export activity. 293T cells were transfected with either GFP or GFP-NXF1 and 48 h later infected with the panel of WT and NS1 mutant viruses. At 6 h p.i., total RNA was extracted before or after the lysates had been subjected to GFP-Trap pulldown, and primer extension reactions were carried out to assay segment 7 mRNAs and vRNA, as well as 5S rRNA as a loading control. Viral RNAs were detected in the total fraction of every infected sample (Fig. 9B, lanes 1 to 7) but only 5S rRNA was detected in mock-infected cells (Fig. 9B, lane 8). Analysis of the bound fractions from WT virus-infected cells showed that both segment 7 mRNA species coprecipitated with GFP-NXF1 (lane 10), agreeing with a previous study (15). This interaction was specific since genomic vRNA did not precipitate with GFP-NXF1 (lane 10) while none of the viral RNA species bound to GFP only (lane 9). A similar outcome was obtained with the export-competent NS1 (S+I) mutant virus (lane 15). However, only trace quantities of M1 or M2 mRNAs bound NXF1 in cells infected with the N81 or R+K NS1 mutant virus (lanes 11 and 14), while reduced amounts coprecipitated from cells infected with the single RNA-binding domain R38 and K41 mutants (lanes 12 and 13). Thus, the ability of NS1 polypeptides to direct efficient export of segment 7 mRNA showed a strong correlation with their ability to promote the interaction of the transcripts with NXF1, consistent with the adaptor protein hypothesis.

**FIG 10:**
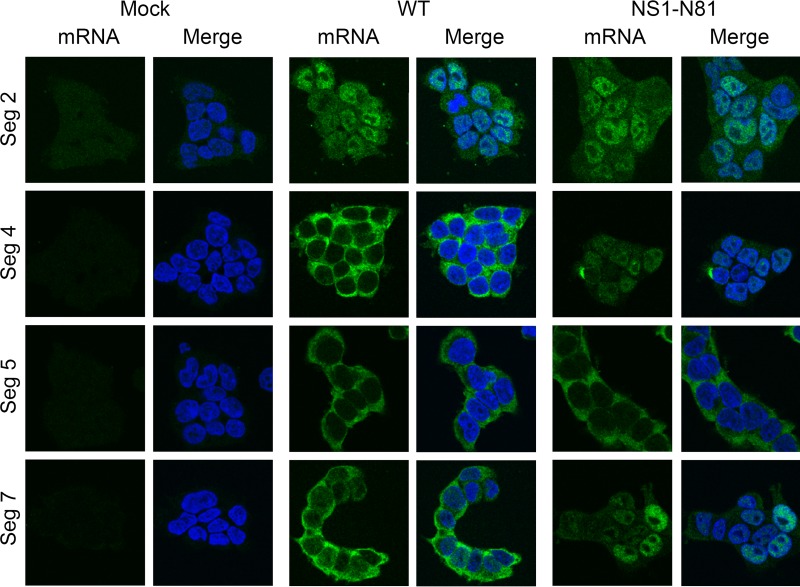
Role of NS1 in promoting export of other viral mRNAs. 293T cells were infected or mock infected with the indicated reverse genetics PR8 viruses at an MOI of 5, fixed at 6 h p.i., and stained for positive-sense RNAs from the indicated segments (green) and for DNA (4′,6′-diamidino-2-phenylindole; blue) before confocal imaging. Single optical slices are shown. Scale bar, 10 μm.

Finally, we asked if NS1 plays a similar role in promoting the nuclear export of other viral mRNAs. Prior studies of nuclear export and/or expression indicated that the other late gene mRNAs for HA and NA show sensitivities similar to those of the M gene mRNAs to treatment with inhibitors of RNA Pol II, whereas RNP genes belonging to the early class do not (7, 8, 12, 15, 39, 40). We therefore compared the intracellular localization of the PB1 polymerase gene (segment 2), NP (segment 5), and HA (segment 4) mRNA in cells infected with the WT or the NS1 N81 mutant virus by FISH. Positive-sense segment 5 RNA was almost exclusively detected in the cytoplasm of cells infected with either virus (Fig. 10), indicating that, unlike segment 7 mRNAs, NP mRNA nuclear export does not require an intact NS1 polypeptide. Segment 2-specific signal was most prominent in the nuclei of infected cells, perhaps reflecting proportionally greater detection of cRNA from a segment where the two classes of positive-sense RNA are present in similar amounts (41, 42). However, the levels of cytoplasmic staining seen in the WT infection were not noticeably diminished in cells infected with the NS1-N81 virus, suggesting that segment 2 mRNA export is also NS1 independent. In contrast, HA mRNA localization altered from being almost totally cytoplasmic in WT infected cells to marked nuclear retention in NS1-N81-infected cells. Thus, NS1 has a role in promoting the efficient nuclear export of viral late gene mRNAs.

## DISCUSSION

The influenza A virus NS1 protein is a polyfunctional molecule that exerts many positive effects on the virus life cycle though a multitude of interactions with other viral and cellular molecules (32). Here, we characterize a novel functional role of NS1 in promoting the nuclear export of viral late gene mRNAs, in particular, M1 but also HA mRNA. We base this conclusion on the notable difference in the bulk localizations of segment 7 mRNA between infected and RNP-transfected cells, a difference that could be obviated by the additional expression of NS1, supported further by the similar alterations in segment 7 mRNA localization seen in cells infected with NS1 mutant viruses. We also found that NS1 increased the amount of segment 7 mRNA bound by NXF1 and that there was a good correlation between the ability of NS1 mutants to perform this function and to bind NXF1 itself, suggesting that NS1 acts as an adaptor protein providing a bridge between the viral transcription machinery and the cellular mRNA export pathway. In this model, NS1 replaces (or perhaps augments) the role of the cellular protein Aly in recruiting the NXF1/p15 complex, thus sidestepping potential blocks to the recruitment of the mRNA export machinery arising from the lack of exon-junction complex deposition or competition between the cellular cap-binding complex and the viral polymerase, both of which are normal routes to attract Aly (17, 18, 43).

Our results are broadly consistent with and complement those recently reported by Mor and colleagues regarding the role of NS1 in the nuclear export of segment 7 mRNAs, with our study focusing on the role of NS1 and theirs focusing more on NS1-mediated intranuclear trafficking of segment 7 mRNA to nuclear speckles for splicing (23). We agree on the point that, ultimately, NS1 promotes nuclear export of unspliced M1 mRNA and extend the finding by showing that both the RNA-binding and effector domains of the protein are needed for the activity, which correlates with the ability to act as a bridge between NXF1 and the viral transcript. However, we do not find that this role of NS1 necessarily correlates with a major effect on transcript splicing. While cells infected with the N81 NS1 mutant produced lower levels of mRNA2 than WT virus-infected cells (Fig. 6), consistent with the effects observed by Mor et al., we did not see a consistent effect of NS1 in a minireplicon setting (Fig. 3), and we also show that NS1 promotes nuclear export of the intronless HA transcript (Fig. 10).

A role for NS1 in viral mRNA nuclear export is consistent with its known interaction with viral RNPs (44, 45), which would place it in the correct location to interact with a newly synthesized viral mRNA. The association between the viral polymerase and cellular RNA Pol II may also serve to place the nascent viral transcripts in the vicinity of the cellular nuclear export apparatus (14, 46). The interaction of NS1 with the NXF1 export apparatus has also been proposed as a mechanism by which the virus inhibits the export of cellular mRNAs (36). Although the mechanism by which NS1 inhibits cellular mRNA export by blocking 3′-end processing and polyadenylation has been described (47), it is less obvious how targeting the mRNA export apparatus could distinguish viral from cellular mRNAs. One possibility might be via time-dependent effects: an early positive effect on viral mRNA export followed by late inhibition of the export pathway.

Although NS1 promotes the cytoplasmic accumulation of M1 mRNA, it is not absolutely required since reduced amounts of cytoplasmic transcripts were still detectable (Fig. 2C) and translated even in the absence of the protein (Fig. 2D). This is consistent with the fundamental observation that NS1 is a nonessential viral gene in cell culture whose loss nevertheless attenuates virus replication even in interferon-deficient systems (48). The 3- to 4-fold reduction in M1 accumulation we saw is consistent with the decreases in late gene expression noted in many previous studies of NS1 mutants (29, 49–53). The low-efficiency export of M1 mRNA in the absence of NS1 may be attributable to direct recruitment of the export apparatus through interactions with the nuclear cap-binding complex (14). A degree of redundancy in the cellular factors recruited for viral mRNA export has precedents from other viral systems (54).

Cell-to-cell variability in nuclear export of segment 7 mRNA in the absence of an intact NS1 protein was also notable. A minority of cells showed cytoplasmic mRNA even in the absence of NS1 (Fig. 2B), and NS1 mutants with an overall defect in promoting cytoplasmic accumulation of M1 mRNA produced an altered ratio of cells with apparently normal export to those displaying nuclear retention (Fig. 4 and 5) instead of an equally reduced efficiency of mRNA cytoplasmic accumulation in all cells. The reasons for this are not obvious, but we conjecture that these observations reflect cell-to-cell variability rather than viral heterogeneity because we saw similar outcomes from plasmid- and viral-based systems (e.g., compare the R+K mutants in Fig. 4B and 5B). In some respects, this heterogeneity in mRNA export is reminiscent of the cell-to-cell variability seen in the triggering of innate responses in negative-sense RNA virus-infected cells (55). Given the central role of NS1 in counteracting innate responses and the evidence linking this to direct effects on the cellular nuclear export apparatus (32, 36, 56), this is an aspect that warrants further investigation.

## MATERIALS AND METHODS

### Cells, plasmids, antisera, and viruses.

Human embryonic kidney 293T cells and human alveolar basal epithelial cells (A549) were cultured as described previously (57). Plasmids pcDNA-PB2, pcDNA-PB1, pcDNA-PA, and pcDNA-NP used for the minireplicon assay have been described previously (58), and plasmids pPolI-segment 4, pPolI-segment 7, and pPolI-segment 8 were a gift from Ron Fouchier (59). The NS1 expression vector was kindly provided by Wendy Barclay. NS1 and NS2 genes were PCR cloned into pEGFPN1 in fusion with enhanced GFP (EGFP) using AgeI and KpnI as restriction sites. NS1 mutants N81, R38A, K41A, R38A+K41A (R+K), and S103/106I (S+I) were produced by site-directed mutagenesis (Stratagene). The segment 7 intron was cloned into pcDNA3 (Invitrogen) using EcoRI and HinDIII sites after PCR amplification of the intron sequence using *Pfu* polymerase (Stratagene). Primer sequences are available on request. GFP-NXF1 plasmid was a gift from Adrian Whitehouse (60). The Cambridge lineage of A/PR/8/34 virus was propagated in embryonated eggs as previously described (61). WT and mutant A/PR/8/34 viruses were rescued in 293T cells using an eight-plasmid system (59) as previously described.

### Transfection and infection.

RNP reconstitution assays were carried out by transfecting 293T cells with 135 ng each of plasmids pcDNA-PB2, pcDNA-PB1, pcDNA-PA, and pcDNA-NP (3PNP), pPolI segment 7, and various others as required by the experimental design. At 24 h posttransfection (hpt) samples were processed as described previously (58). Cells were transfected using Lipofectamine 2000 (Invitrogen) according to the manufacturer's instructions. Alternatively, 293T cells were infected with wild-type A/PR/8/34 and NS1 mutant viruses at a multiplicity of infection (MOI) of 5, and at 6 h postinfection p.i. samples were processed as described in Amorim et al. (8).

### FISH, GFP-Trap pulldown, primer extensions, and protein analysis.

FISH analysis was performed as described previously (8, 12) at either 24 hpt or 6 h p.i. RNA probes used to detect segment 7 mRNA were labeled using cyanine 3-UTP (PerkinElmer) or ChromaTide Alexa Fluor 488-5-UTP (Invitrogen) as previously described (8, 12). GFP-Trap pulldown assays were carried out by transfecting 293T cells with 2 μg of a GFP-tagged plasmid (NXF1, WT NS1, or NS1 mutants) and with 250 ng of pEGFPN1 plasmid used as a negative control. Cells were superinfected at 48 hpt with either WT A/PR/8/34 or an NS1 mutant virus at an MOI of 10 and at 6 h p.i. collected for the pulldown assays performed using GFP-Trap beads (Chromatek) as previously described (62). In minireplicon transfections followed by GFP-Trap pulldown assays, 500 ng of each plasmid was transfected along with a WT or mutant NS1 GFP-tagged plasmid. The bound fractions of samples were either boiled in SDS-PAGE sample buffer to study protein-protein interactions or used for RNA extraction to analyze protein-RNA interactions. Bound RNA species were identified by reverse transcriptase radiolabeled-primer extension followed by urea-PAGE and autoradiography as described previously (42). Protein analysis was performed by SDS-PAGE and Western blotting according to standard procedures. Blots were imaged using a Licor Biosciences Odyssey near-infrared imaging platform. Protein quantifications were performed using Licor Odyssey, version 3, software.

## References

[1] Te Velthuis AJ, Fodor E 2016 Influenza virus RNA polymerase: insights into the mechanisms of viral RNA synthesis. Nat Rev Microbiol 14:479–493. doi:10.1038/nrmicro.2016.87. 27396566 PMC4966622

[2] Hutchinson EC, Fodor E 2013 Transport of the influenza virus genome from nucleus to nucleus. Viruses 5:2424–2446. doi:10.3390/v5102424. 24104053 PMC3814596

[3] Boulo S, Akarsu H, Ruigrok RW, Baudin F 2007 Nuclear traffic of influenza virus proteins and ribonucleoprotein complexes. Virus Res 124:12–21. doi:10.1016/j.virusres.2006.09.013. 17081640

[4] Dubois J, Terrier O, Rosa-Calatrava M 2014 Influenza viruses and mRNA splicing: doing more with less. mBio 5:e00070-14. doi:10.1128/mBio.00070-14. 24825008 PMC4030477

[5] Inglis SC, Carroll AR, Lamb RA, Mahy BW 1976 Polypeptides specified by the influenza virus genome. I. Evidence for eight distinct gene products specified by fowl plague virus. Virology 74:489–503. 982839 10.1016/0042-6822(76)90355-x

[6] Dolan BP, Knowlton JJ, David A, Bennink JR, Yewdell JW 2010 RNA polymerase II inhibitors dissociate antigenic peptide generation from normal viral protein synthesis: a role for nuclear translation in defective ribosomal product synthesis? J Immunol 185:6728–6733. doi:10.4049/jimmunol.1002543. 21048111 PMC3398797

[7] Vogel U, Kunerl M, Scholtissek C 1994 Influenza A virus late mRNAs are specifically retained in the nucleus in the presence of a methyltransferase or a protein kinase inhibitor. Virology 198:227–233. doi:10.1006/viro.1994.1025. 8259658

[8] Amorim MJ, Read EK, Dalton RM, Medcalf L, Medcalf L, Digard P 2007 Nuclear export of influenza A virus mRNAs requires ongoing RNA polymerase II activity. Traffic 8:1–11. doi:10.1111/j.1600-0854.2006.00507.x. 17132145

[9] Erkmann JA, Kutay U 2004 Nuclear export of mRNA: from the site of transcription to the cytoplasm. Exp Cell Res 296:12–20. doi:10.1016/j.yexcr.2004.03.015. 15120988

[10] Herold A, Suyama M, Rodrigues JP, Braun IC, Kutay U, Carmo-Fonseca M, Bork P, Izaurralde E 2000 TAP (NXF1) belongs to a multigene family of putative RNA export factors with a conserved modular architecture. Mol Cell Biol 20:8996–9008. doi:10.1128/MCB.20.23.8996-9008.2000. 11073998 PMC86553

[11] Hao L, Sakurai A, Sakurai A, Watanabe T, Sorensen E, Sorensen E, Nidom CA, Newton MA, Ahlquist P, Kawaoka Y 2008 *Drosophila* RNAi screen identifies host genes important for influenza virus replication. Nature 454:890–893. doi:10.1038/nature07151. 18615016 PMC2574945

[12] Read EKC, Digard P 2010 Individual influenza A virus mRNAs show differential dependence on cellular NXF1/TAP for their nuclear export. J Gen Virol 91:1290–1301. doi:10.1099/vir.0.018564-0. 20071484 PMC3052562

[13] Larsen S, Bui S, Perez V, Mohammad A, Medina-Ramirez H, Newcomb LL 2014 Influenza polymerase encoding mRNAs utilize atypical mRNA nuclear export. Virol J 11:154. doi:10.1186/1743-422X-11-154. 25168591 PMC4158059

[14] Bier K, York A, Fodor E 2011 Cellular cap-binding proteins associate with influenza virus mRNAs. J Gen Virol 92:1627–1634. doi:10.1099/vir.0.029231-0. 21402597

[15] Wang W, Cui Z-Q, Han H, Zhang Z-P, Wei H-P, Zhou Y-F, Chen Z, Zhang X-E 2008 Imaging and characterizing influenza A virus mRNA transport in living cells. Nucleic Acids Res 36:4913–4928. doi:10.1093/nar/gkn475. 18653528 PMC2528172

[16] Masuda S, Das R, Cheng H, Hurt E, Dorman N, Reed R 2005 Recruitment of the human TREX complex to mRNA during splicing Genes Dev 19:1512–1517. 15998806 10.1101/gad.1302205 PMC1172058

[17] Cheng H, Dufu K, Lee C-S, Hsu JL, Dias A, Reed R 2006 Human mRNA export machinery recruited to the 5′ end of mRNA. Cell 127:1389–1400. doi:10.1016/j.cell.2006.10.044. 17190602

[18] Nojima T, Hirose T, Kimura H, Hagiwara M 2007 The interaction between cap-binding complex and RNA export factor is required for intronless mRNA export. J Biol Chem 282:15645–15651. doi:10.1074/jbc.M700629200. 17363367

[19] Strässer K, Masuda S, Mason P, Pfannstiel J, Oppizzi M, Rodriguez-Navarro S, Rondón AG, Aguilera A, Struhl K, Reed R, Hurt E 2002 TREX is a conserved complex coupling transcription with messenger RNA export. Nature 417:304–308. doi:10.1038/nature746. 11979277

[20] Hautbergue GM, Hung ML, Golovanov AP, Lian LY, Wilson SA 2008 Mutually exclusive interactions drive handover of mRNA from export adaptors to TAP. Proc Natl Acad Sci U S A 105:5154–5159. doi:10.1073/pnas.0709167105. 18364396 PMC2278192

[21] Boyne JR, Colgan KJ, Whitehouse A 2008 Recruitment of the complete hTREX complex is required for Kaposi's sarcoma-associated herpesvirus intronless mRNA nuclear export and virus replication. PLoS Pathog 4:e1000194. doi:10.1371/journal.ppat.1000194. 18974867 PMC2569588

[22] Schneider J, Wolff T 2009 Nuclear functions of the influenza A and B viruses NS1 proteins: do they play a role in viral mRNA export? Vaccine 27:6312–6316. doi:10.1016/j.vaccine.2009.01.015. 19840666

[23] Mor A, White A, Zhang K, Thompson M, Esparza M, Muñoz-Moreno R, Koide K, Lynch KW, García-Sastre A, Fontoura BMA 2016 Influenza virus mRNA trafficking through host nuclear speckles. Nat Microbiol 1:16069. doi:10.1038/nmicrobiol.2016.69. 27572970 PMC4917225

[24] Lamb RA, Lai CJ 1980 Sequence of interrupted and uninterrupted mRNAs and cloned DNA coding for the two overlapping nonstructural proteins of influenza virus. Cell 21:475–485. doi:10.1016/0092-8674(80)90484-5. 7407920

[25] Inglis SC, Barrett T, Brown CM, Almond JW 1979 The smallest genome RNA segment of influenza virus contains two genes that may overlap. Proc Natl Acad Sci U S A 76:3790–3794. doi:10.1073/pnas.76.8.3790. 291039 PMC383920

[26] Inglis SC, Brown CM 1981 Spliced and unspliced RNAs encoded by virion RNA segment 7 of influenza virus. Nucleic Acids Res 9:2727–2740. doi:10.1093/nar/9.12.2727. 6169001 PMC326888

[27] Lamb RA, Lai CJ, Choppin PW 1981 Sequences of mRNAs derived from genome RNA segment 7 of influenza virus: colinear and interrupted mRNAs code for overlapping proteins. Proc Natl Acad Sci U S A 78:4170–4174. doi:10.1073/pnas.78.7.4170. 6945577 PMC319750

[28] Holsinger LJ, Shaughnessy MA, Lamb RA 1995 Analysis of the posttranslational modifications of the influenza virus M2 protein. J Virol 69:1219–1225. 7529332 10.1128/jvi.69.2.1219-1225.1995 PMC188695

[29] Salvatore M, Basler CF, Parisien J-P, Horvath CM, Bourmakina S, Zheng H, Muster T, Palese P, García-Sastre A 2002 Effects of influenza A virus NS1 protein on protein expression: the NS1 protein enhances translation and is not required for shutoff of host protein synthesis. J Virol 76:1206–1212. doi:10.1128/JVI.76.3.1206-1212.2002. 11773396 PMC135795

[30] Lu Y, Qian XY, Krug RM 1994 The influenza virus NS1 protein: a novel inhibitor of pre-mRNA splicing. Genes Dev 8:1817–1828. doi:10.1101/gad.8.15.1817. 7958859

[31] Robb NC, Fodor E 2012 The accumulation of influenza A virus segment 7 spliced mRNAs is regulated by the NS1 protein. J Gen Virol 93:113–118. doi:10.1099/vir.0.035485-0. 21918006

[32] Hale BG, Randall RE, Ortín J, Jackson D 2008 The multifunctional NS1 protein of influenza A viruses. J Gen Virol 89:2359–2376. doi:10.1099/vir.0.2008/004606-0. 18796704

[33] Kochs G, García-Sastre A, Martínez-Sobrido L 2007 Multiple anti-interferon actions of the influenza A virus NS1 protein. J Virol 81:7011–7021. doi:10.1128/JVI.02581-06. 17442719 PMC1933316

[34] Kuo R-L, Krug RM 2009 Influenza a virus polymerase is an integral component of the CPSF30-NS1A protein complex in infected cells. J Virol 83:1611–1616. doi:10.1128/JVI.01491-08. 19052083 PMC2643760

[35] Kaida D, Motoyoshi H, Tashiro E, Nojima T, Hagiwara M, Ishigami K, Watanabe H, Kitahara T, Yoshida T, Nakajima H, Tani T, Horinouchi S, Yoshida M 2007 Spliceostatin A targets SF3b and inhibits both splicing and nuclear retention of pre-mRNA. Nat Chem Biol 3:576–583. doi:10.1038/nchembio.2007.18. 17643111

[36] Satterly N, Tsai PL, van Deursen J, Nussenzveig DR, Wang Y, Faria PA, Levay A, Levy DE, Fontoura BM 2007 Influenza virus targets the mRNA export machinery and the nuclear pore complex. Proc Natl Acad Sci U S A 104:1853–1858. doi:10.1073/pnas.0610977104. 17267598 PMC1794296

[37] Park YW, Katze MG 1995 Translational control by influenza virus. Identification of cis-acting sequences and trans-acting factors which may regulate selective viral mRNA translation. J Biol Chem 270:28433–28439. 7499349 10.1074/jbc.270.47.28433

[38] Burgui I, Aragón T, Ortín J, Nieto A 2003 PABP1 and eIF4GI associate with influenza virus NS1 protein in viral mRNA translation initiation complexes. J Gen Virol 84:3263–3274. doi:10.1099/vir.0.19487-0. 14645908

[39] Kurokawa M, Ochiai H, Nakajima K, Niwayama S 1990 Inhibitory effect of protein kinase C inhibitor on the replication of influenza type A virus. J Gen Virol 71:2149–2155. doi:10.1099/0022-1317-71-9-2149. 1698925

[40] Kistner O, Müller K, Scholtissek C 1989 Differential phosphorylation of the nucleoprotein of influenza A viruses. J Gen Virol 70:2421–2431. doi:10.1099/0022-1317-70-9-2421. 2778438

[41] Dalton RM, Mullin AE, Amorim MJ, Medcalf E, Tiley LS, Digard P 2006 Temperature sensitive influenza A virus genome replication results from low thermal stability of polymerase-cRNA complexes. Virol J 3:58. doi:10.1186/1743-422X-3-58. 16934156 PMC1569369

[42] Robb NC, Smith M, Vreede FT, Fodor E 2009 NS2/NEP protein regulates transcription and replication of the influenza virus RNA genome. J Gen Virol 90:1398–1407. doi:10.1099/vir.0.009639-0. 19264657

[43] Taniguchi I, Ohno M 2008 ATP-dependent recruitment of export factor Aly/REF onto intronless mRNAs by RNA helicase UAP56. Mol Cell Biol 28:601–608. doi:10.1128/MCB.01341-07. 17984224 PMC2223434

[44] Marion RM, Zurcher T, la Luna de S, Ortin J 1997 Influenza virus NS1 protein interacts with viral transcription-replication complexes in vivo. J Gen Virol 78:2447–2451. doi:10.1099/0022-1317-78-10-2447. 9349463

[45] Robb NC, Chase G, Bier K, Vreede FT, Shaw P-C, Naffakh N, Schwemmle M, Abbas MA, Fodor E 2011 The influenza A virus NS1 protein interacts with the nucleoprotein of viral ribonucleoprotein complexes. J Virol 85:5228–5231. doi:10.1128/JVI.02562-10. 21411538 PMC3126214

[46] Engelhardt OG, Smith M, Fodor E 2005 Association of the influenza A virus RNA-dependent RNA polymerase with cellular RNA polymerase II. J Virol 79:5812–5818. doi:10.1128/JVI.79.9.5812-5818.2005. 15827195 PMC1082766

[47] Nemeroff ME, Barabino SM, Li Y, Keller W, Krug RM 1998 Influenza virus NS1 protein interacts with the cellular 30 kDa subunit of CPSF and inhibits 3′ end formation of cellular pre-mRNAs. Mol Cell 1:991–1000. doi:10.1016/S1097-2765(00)80099-4. 9651582

[48] García-Sastre A, Egorov A, Matassov D, Brandt S, Levy DE, Durbin JE, Palese P, Muster T 1998 Influenza A virus lacking the NS1 gene replicates in interferon-deficient systems. Virology 252:324–330. doi:10.1006/viro.1998.9508. 9878611

[49] Egorov A, Brandt S, Sereinig S, Romanova J, Ferko B, Katinger D, Grassauer A, Alexandrova G, Katinger H, Muster T 1998 Transfectant influenza A viruses with long deletions in the NS1 protein grow efficiently in Vero cells. J Virol 72:6437–6441. 9658085 10.1128/jvi.72.8.6437-6441.1998 PMC109801

[50] Hatada E, Hasegawa M, Shimizu K, Hatanaka M, Fukuda R 1990 Analysis of influenza A virus temperature-sensitive mutants with mutations in RNA segment 8. J Gen Virol 71:1283–1292. doi:10.1099/0022-1317-71-6-1283. 2141068

[51] Enami M, Enami K 2000 Characterization of influenza virus NS1 protein by using a novel helper-virus-free reverse genetic system. J Virol 74:5556–5561. doi:10.1128/JVI.74.12.5556-5561.2000. 10823862 PMC112042

[52] Falcón AM, Marión RM, Zürcher T, Gómez P, Portela A, Nieto A, Ortín J 2004 Defective RNA replication and late gene expression in temperature-sensitive influenza viruses expressing deleted forms of the NS1 protein. J Virol 78:3880–3888. doi:10.1128/JVI.78.8.3880-3888.2004. 15047804 PMC374278

[53] Garaigorta U, Falcón AM, Ortín J 2005 Genetic analysis of influenza virus NS1 gene: a temperature-sensitive mutant shows defective formation of virus particles. J Virol 79:15246–15257. doi:10.1128/JVI.79.24.15246-15257.2005. 16306596 PMC1316024

[54] Jackson BR, Boyne JR, Noerenberg M, Taylor A, Hautbergue GM, Walsh MJ, Wheat R, Blackbourn DJ, Wilson SA, Whitehouse A 2011 An interaction between KSHV ORF57 and UIF provides mRNA-adaptor redundancy in herpesvirus intronless mRNA export. PLoS Pathog 7:e1002138. doi:10.1371/journal.ppat.1002138. 21814512 PMC3141038

[55] Chen S, Short JA, Young DF, Killip MJ, Schneider M, Goodbourn S, Randall RE 2010 Heterocellular induction of interferon by negative-sense RNA viruses. Virology 407:247–255. doi:10.1016/j.virol.2010.08.008. 20833406 PMC2963793

[56] Mata MA, Satterly N, Versteeg GA, Frantz D, Wei S, Williams N, Schmolke M, Peña-Llopis S, Brugarolas J, Forst CV, White MA, Garcia-Sastre A, Roth MG, Fontoura BM 2011 Chemical inhibition of RNA viruses reveals REDD1 as a host defense factor. Nat Chem Biol 7:712–719. doi:10.1038/nchembio.645. 21909097 PMC3329801

[57] Carrasco M, Amorim MJ, Digard P 2004 Lipid raft-dependent targeting of the influenza A virus nucleoprotein to the apical plasma membrane. Traffic 5:979–992. doi:10.1111/j.1600-0854.2004.00237.x. 15522099

[58] Mullin AE, Dalton RM, Amorim MJ, Elton D, Digard P 2004 Increased amounts of the influenza virus nucleoprotein do not promote higher levels of viral genome replication. J Gen Virol 85:3689–3698. doi:10.1099/vir.0.80518-0. 15557242

[59] de Wit E, Spronken MI, Bestebroer TM, Rimmelzwaan GF, Osterhaus AD, Fouchier RA 2004 Efficient generation and growth of influenza virus A/PR/8/34 from eight cDNA fragments. Virus Res 103:155–161. doi:10.1016/j.virusres.2004.02.028. 15163504

[60] Williams BJ, Boyne JR, Goodwin DJ, Roaden L, Hautbergue GM, Wilson SA, Whitehouse A 2005 The prototype gamma-2 herpesvirus nucleocytoplasmic shuttling protein, ORF 57, transports viral RNA through the cellular mRNA export pathway. Biochem J 387:295–308. doi:10.1042/BJ20041223. 15537388 PMC1134957

[61] Elton D, Simpson-Holley M, Archer K, Medcalf L, Hallam R, McCauley J, Digard P 2001 Interaction of the influenza virus nucleoprotein with the cellular CRM1-mediated nuclear export pathway. J Virol 75:408–419. doi:10.1128/JVI.75.1.408-419.2001. 11119609 PMC113933

[62] Amorim MJ, Bruce EA, Read EKC, Foeglein A, Mahen R, Stuart AD, Digard P 2011 A Rab11- and microtubule-dependent mechanism for cytoplasmic transport of influenza A virus viral RNA. J Virol 85:4143–4156. doi:10.1128/JVI.02606-10. 21307188 PMC3126276

